# Harnessing of Artificial Intelligence for the Diagnosis and Prevention of Hospital-Acquired Infections: A Systematic Review

**DOI:** 10.3390/diagnostics14050484

**Published:** 2024-02-23

**Authors:** Buket Baddal, Ferdiye Taner, Dilber Uzun Ozsahin

**Affiliations:** 1 Department of Medical Microbiology and Clinical Microbiology, Faculty of Medicine, Near East University, North Cyprus, Mersin 10, 99138 Nicosia, Turkey; ferdiye.taner@neu.edu.tr; 2 DESAM Research Institute, Near East University, North Cyprus, Mersin 10, 99138 Nicosia, Turkey; 3 Department of Medical Diagnostic Imaging, College of Health Science, University of Sharjah, Sharjah 27272, United Arab Emirates; dilber.uzunozsahin@neu.edu.tr; 4 Research Institute for Medical and Health Sciences, University of Sharjah, Sharjah 27272, United Arab Emirates; 5 Operational Research Centre in Healthcare, Near East University, North Cyprus, Mersin 10, 99138 Nicosia, Turkey

**Keywords:** artificial intelligence, hospital-acquired infections, diagnosis, forecasting, prediction, antimicrobial resistance

## Abstract

Healthcare-associated infections (HAIs) are the most common adverse events in healthcare and constitute a major global public health concern. Surveillance represents the foundation for the effective prevention and control of HAIs, yet conventional surveillance is costly and labor intensive. Artificial intelligence (AI) and machine learning (ML) have the potential to support the development of HAI surveillance algorithms for the understanding of HAI risk factors, the improvement of patient risk stratification as well as the prediction and timely detection and prevention of infections. AI-supported systems have so far been explored for clinical laboratory testing and imaging diagnosis, antimicrobial resistance profiling, antibiotic discovery and prediction-based clinical decision support tools in terms of HAIs. This review aims to provide a comprehensive summary of the current literature on AI applications in the field of HAIs and discuss the future potentials of this emerging technology in infection practice. Following the PRISMA guidelines, this study examined the articles in databases including PubMed and Scopus until November 2023, which were screened based on the inclusion and exclusion criteria, resulting in 162 included articles. By elucidating the advancements in the field, we aim to highlight the potential applications of AI in the field, report related issues and shortcomings and discuss the future directions.

## 1. Introduction

With the major challenges healthcare systems have encountered worldwide with the rapid increase in the number of patients and excessive workload in diagnostic laboratories during the coronavirus disease (COVID-19) pandemic, the implementation of automation and machine learning (ML) has become more and more important in the field of infectious diseases. In the clinical context, the interpretation of laboratory results by highly trained microbiologists and/or laboratory personnel is critical, as these specimens provide important clinical and diagnostic information which can direct therapy [1]. Therefore, in the shortage of medical laboratory personnel, the use of artificial intelligence (AI) applications for the automation of experimentally demanding and visually interpretative tasks offer an advantage for the workflow in a clinical microbiology laboratory [2]. AI has been also thoroughly investigated in terms of its application to the prediction and prevention on infectious diseases in the healthcare settings. Hospital-acquired infections (HAIs), defined as infections occurring during the process of care, are a global public health concern and represent the most frequent adverse events in healthcare with an estimated over 2.6 million new cases of HAIs happening every year in Europe [3]. Considering the alarming problem of antimicrobial resistance worldwide and the high incidence of HAIs with high mortality and morbidity rates, forecasting infectious diseases in hospitals and intensive care units (ICUs) by exploiting AI applications has become fundamental. While the surveillance of HAIs represents the foundation for organizing, implementing and maintaining effective infection prevention and control programs, AI has become a valuable tool applicable in this context within healthcare facilities yet faces some challenges [4] (Table 1). The overarching aim of this review is to give a comprehensive overview of AI applications in the field of microbiology and the latest developments in the area of diagnosis and prevention of HAIs.

## 2. Materials and Methods

### 2.1. Search Strategy

In accordance with the Preferred Reporting Items for Systematic Reviews and Meta-Analyses (PRISMA) guidelines [5], a systematic search was conducted using the databases PubMed and Scopus to find potentially eligible articles. Search strategies focused on two main concepts of the use of AI (using “machine learning”, “deep learning”, “artificial intelligence”, “automated”) in (1) diagnostic microbiology using the following terms: “COVID-19”, “imaging”, “RT-PCR”, “CT”, “X-ray”, “radiograph”, “ultrasound”, “diagnostic”, “clinical”, “microbiology”, “laboratory”, “bacteria”, “virus”, “fungus”, “parasitic”, “parasite”, “image”, “microscopy”, “culture”, “smear”, “microscopy”, “microbiology”, “colony”, “MALDI-TOF”, “Raman”, “SERS”, whole genome sequencing”, “molecular typing”, “factor analysis”, “blood parameters”, “urine parameters”, “antibiotic resistance”, “resistance genes”, “resistant bacteria”, “antibiotic discovery”, “screening”, “peptides”, “chemical library”, “microbiome”, “metagenomics”, “metatranscriptomics”, “metabolomics”; (2) in hospital-acquired infections (HAIs) using the following terms: “intensive care unit”, infection”, “prediction”, “forecasting”, “risk factors”, “patient risk stratification”, “real-time detection”, “HAI surveillance”, “HAI monitoring”, “clinical decision support”, “ventilator associated pneumonia”, “central line associated bloodstream infections”, “surgical site infections”, “sepsis”, “*Clostridium difficile*”, “multi-drug resistant”, and “hand-hygiene”. No limitations were applied.

### 2.2. Study Selection

Original studies that assessed the application of machine learning in the surveillance, diagnosis, prediction and prevention of infectious diseases and hospital-acquired infections until November 2023 were considered eligible for inclusion. The exclusion criteria consisted of commentary articles, correspondence, letters to the editor, conference abstracts, editorials, and non-English language publications. Research that poses a significant risk of bias such as M.Sc. and Ph.D. theses, seminars, and posters were also excluded. To find more pertinent articles, the reference lists of the chosen articles were screened. Review articles were also obtained in order to find more potential studies. Original articles for the ‘COVID-19’ section of the manuscript included only those which were retrieved using “COVID-19” and “RT-PCR”, as this was the gold standard for microbiological diagnosis. For this section, review articles were selected using “COVID-19”, “CT”, “X-ray”, “radiograph”, “ultrasound” and only selected comprehensive papers were mentioned in the manuscript, as this was not the scope of the current review.

The titles and abstracts of all obtained studies were independently screened by two reviewers (B.B. and F.T., associate and assistant professors in Medical and Clinical Microbiology with >10 years of research experience in infectious diseases). After the exclusion of the duplicate studies, the full text of all eligible articles was assessed. All discrepancies were addressed, and a mutual consensus was reached among the authors regarding the final inclusion.

### 2.3. Data Extraction and Assessment of Quality

Authors’ names, years, and descriptive data of all studies including sample size, study design, techniques, parameters, and the subject matter of each study were extracted. The following characteristic data were also obtained if they were provided: the analysis tool that was used for the study, including methods like regression models, LASSO, XGBoost, and deep learning, convolutional neural networks; the performance measures, including the area under the receiver operator curve (AUROC), accuracy, sensitivity, specificity, number of variables included, and conclusion. The risk of bias in the selected studies was assessed by two reviewers (B.B. and F.T.). Any disagreement regarding the judge was handled or discussed with a third researcher (DUO). 

## 3. Results

**Figure 1:**
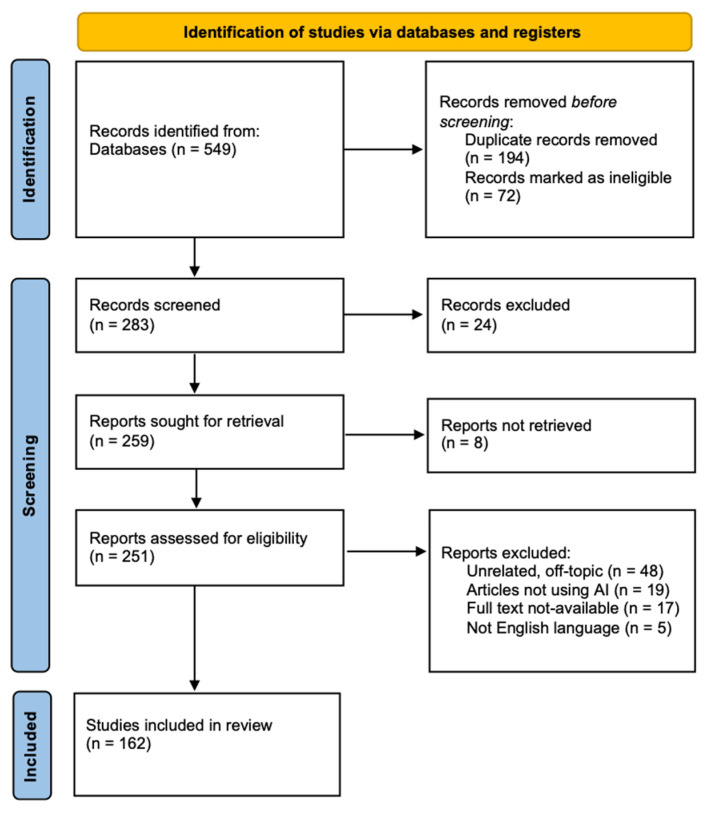
PRISMA flow diagram for the search process.

A total of 549 articles were found in the aforementioned databases after the searches, of which 194 were excluded as they were duplicates or were marked as ineligible. The initial screening of the remaining 283 articles by title and abstract resulted in the exclusion of 24 that did not meet the inclusion criteria of the current review. Among the remaining 259 articles, 8 of them were excluded by two reviewers. A total of 251 articles were analyzed and 89 were excluded for different reasons such as being unrelated and off-topic, not using AI algorithms, being not in English language and full-text not being available. Finally, 162 articles met the inclusion criteria and were included in the current systematic review. Figure 1 illustrated the PRISMA flow diagram for the search process.

### 3.1. AI Applications in Microbiology

#### 3.1.1. COVID-19

The gold standard for the diagnosis of COVID-19 is the detection of the causative agent, severe acute respiratory syndrome coronavirus 2 (SARS-CoV-2), using a virus-specific reverse transcriptase polymerase chain reaction (RT–PCR) test [6]. With the rapid propagation of COVID-19 due to widespread person-to-person transmission during the pandemic, an unprecedented necessity for PCR testing in diagnostic laboratories worldwide was observed. In order to alleviate the heavy burden of healthcare and laboratory personnel, a number of AI-aided detection models have been developed for the rapid and reliable diagnosis of SARS-CoV-2 via RT-PCR. Alouani et al. proposed a deep learning model, qPCRdeepNet, that utilizes a deep convolutional neural network for the analysis of the fluorescent readings obtained during COVID-19 RT-PCR, which was indicated to detect false positive results and improve test specificity [7]. Lee et al. also developed a deep learning model trained with the long-term short memory (LSTM) method, in which the raw data of fluorescence values in each 40 cycles of RT-PCR test were used. The authors reported a shortened RT-PCR diagnosis time when evaluated with patient’s clinical characteristics, blood test results and chest CT imaging data [8]. Similarly, an AI-based detection and classification system for COVID-19 RT-PCR diagnosis using fluorescent data and amplification curves was developed in which the authors were able to automatically categorize RT-PCR data as positive, weak-positive, negative or re-run. [9]. Moreover, Villarreal-González et al. analyzed 4230 RT-PCR curves from patient data using different ML models, categorized them into positive, early, no and abnormal amplifications, and indicated that the best model was able to detect atypical profiles in PCR curves due to the contamination or artifacts, thereby enabling rapid diagnosis and reducing false positives [10]. The detection of SARS-CoV-2 variants has also been approached. Alvargonzález et al. investigated an ML algorithm based on the number of cycles (cycle threshold, Ct) in RT-PCR data obtained during the pandemic and suggested that the distinguishable patterns in the Ct values of PCR-positive samples can aid in the detection of various virus variants [11]. Similarly, Beduk et al. has applied a Dense Neural Network (DNN) algorithm for the detection of SARS-CoV-2 variants using laser-scribed graphene (LSG) sensors coupled with a gold nanoparticles (AuNPs) biosensing platform [12]. The AI-driven SARS-CoV-2 diagnosis has been invaluable for optimizing the time spent on the analysis of RT-PCR tests and helped to reduce the human intervention required in laboratory practice.

Importantly, a number of studies have focused on the application of ML for the prediction of SARS-CoV-2 positivity using patients’ blood test and serum profiling results. Tschoellitsch et al. trained a Random Forest algorithm using routinely available blood test results with 1353 unique features to predict the RT-PCR test results. The authors reported that the model was able to detect SARS-CoV-2 test results with an accuracy of 81%, an area under the ROC curve of 0.74, a sensitivity of 60%, and a specificity of 82% [13]. Brinati et al., on the other hand, developed two ML classification models using hematochemical values such as white blood cells counts, and the platelets, CRP, AST, ALT, GGT, ALP, and LDH plasma levels from routine blood exams from 279 patients in an attempt to detect COVID-19 infection. The authors reported that both models have an accuracy of 82–86% and sensitivity of 92–95% for the identification of COVID-19 positive patients with respect to the gold standard rRT-PCR [14]. On a similar note, Yang et al. constructed and validated a ML model for COVID-19 diagnosis, identifying the most useful routine blood parameters with high diagnostic accuracy [15]. Abayomi-Alli et al. also compared 15 supervised ML algorithms and applied the ensemble learning approach to develop prediction models for the effective detection of COVID-19 using routine laboratory blood test results [16]. The potential of matrix-assisted laser desorption ionization–time of flight mass spectrometry (MALDI-TOF-MS) combined with ML algorithms was also exploited for the detection of COVID-19 positive and negative protein profiles using nasopharyngeal swab samples [17]. A clinically validated liquid chromatography triple quadrupole method (LC/MS-MS) for the detection of amino acids from plasma specimens has also been described. In this study, targeted plasma metabolomics combined with ML was proven to provide the rapid discrimination of SARS-CoV-2-positive and negative patients [18]. Further studies also focused on the identification of serological signatures of SARS-CoV-2 infection [19], the use of MALDI-TOF-MS, surface-enhanced Raman scattering (SERS), LC-MS, and MALDI-MS for the detection of SARS-CoV-2 in human saliva, nasal swabs, plasma and serum samples [20,21,22,23].

Chest computed tomography (CT) also represents a valuable component of diagnosis in symptomatic patients with suspected SARS-CoV-2 infection and their consequent isolation from the uninfected population [24]. Due to the exponential boost in the number of cases and the similarity of symptoms with other infectious lung diseases, AI has been extensively used in the detection and classification of COVID-19 pneumonia via automated image analysis. A plethora of pioneering studies utilized deep learning for the categorization of chest X-ray (CX-R), CT and ultrasound images to assist radiologists as well as infectious disease specialists with making decisions [25,26,27,28,29,30,31,32,33,34,35,36]. Transfer learning was adopted by Apostolopoulos et al. to a collection of CX-R images including those images with confirmed COVID-19 disease, confirmed common bacterial pneumonia, and images of normal conditions. The authors indicated that the deep learning approach was able to extract significant biomarkers related to the COVID-19 disease in image datasets, while the best accuracy, sensitivity, and specificity obtained was 96.78%, 98.66%, and 96.46%, respectively [25]. Singh and colleagues utilized convolutional neural networks (CNNs) with multi-objective differential evolution (MODE) to analyze chest CT images in an effort to classify the COVID-19-infected patients as infected or not infected, and they indicated that the proposed model can efficiently classify the COVID-19 patients who have abnormalities in chest CT images with most having bilateral multiple lobular and subsegmental areas of consolidation and ground-glass opacity in chest CT images [31]. While chest CT imaging is a valuable component in the evaluation of patients with suspected SARS-CoV-2 infection, CT imaging alone has been indicated to have a limited negative predictive value for ruling out the infection due to normal radiological findings at early stages of the disease. Therefore, Mei et al. have used AI algorithms to integrate chest CT findings with clinical symptoms, history of exposure and laboratory testing to rapidly diagnose COVID-19 positive patients. The authors have reported that the AI system achieved an area under the curve of 0.92 with an equal sensitivity compared to a senior thoracic radiologist [32].

A number of comprehensive systematic reviews on AI-assisted chest imaging for the diagnosis of COVID-19 and other types of pneumonia are also available in literature [37,38,39,40,41,42,43,44]. In particular, diagnosis performance of deep learning models for the interpretation of CT chest scans have been reported to be high. Wang et al., analyzing 51,392 confirmed COVID-19 patients and 7686 non-infected individuals, indicated the pooled sensitivity, the pooled specificity, positive likelihood ratio, negative likelihood ratio and the pooled diagnostic odds ratio (OR) to be 0.87, 0.85, 0.14), and 49, and the AUROC to be 0.94. They also reported that Resnet had the best diagnostic performance with the highest sensitivity of 0.91, specificity of 0.90, and AUROC of 0.96 with the following ranking: Resnet > Densenet > VGG > Mobilenet > Inception > Effficient > Alexnet [42]. Ozsahin et al. provided a review of AI techniques for the diagnosis of COVID-19 with CT imaging and reported the sensitivity, specificity, precision, accuracy, area under the curve, and F1 score results to be as high as 100%, 100%, 99.62, 99.87%, 100%, and 99.5%, respectively, for the categorization of classification tasks including COVID-19/normal, COVID-19/non-COVID-19, COVID-19/non-COVID-19 pneumonia, and severity [44]. As the rapid identification and isolation of SARS-CoV-2 infected individuals was a critical step for the early implementation of preventive interventions, these AI-aided tools have been invaluable for assisting healthcare staff and curbing the spread of the pandemic. The characteristics of the studies are listed in the table below (Table 2).

#### 3.1.2. Image Analysis—Bacterial, Viral, Fungal, Parasitic

Image analysis is a central part of clinical microbiology laboratory diagnostics. Microbiologists are highly trained specialists who can examine and interpret a wide range of clinical diagnostic materials such as Gram stains, fecal, urine and blood smears, which give information regarding the presence of microorganisms and host inflammatory response. Microorganisms also have various phenotypic characteristics and can represent as different growth forms and colors on agar plates when cultured in the laboratory. A variety of solid, semi-solid and liquid chromogenic differential media are utilized by experienced microbiologists to make discriminatory observations when concluding a test result. Due to the experimentally demanding and visually interpretative nature of the work flow, the automation and digitalization of the diagnostic processes using AI represents an important step toward advancing laboratory processes where a shortage of laboratory personnel exists. Overall, the image discrimination capacity of AI has been proposed to increase the efficiency and diagnostic accuracy of clinical microbiology laboratories, which has been thoroughly discussed in a review by Smith et al. Complex, multilayered AI architectures known as deep learning algorithms, of which a subset of these algorithms is known as CNNs, are highly interconnected networks modeled after the human optical cortex that excel at image classification. These algorithms have been used to aid in the diagnostic interpretation of image data to detect infection-related markers, bacterial, fungal and parasitic cellular structures in clinical blood and urine smear specimens, Gram stains, agar plate, chromogenic media, colony and microscopic morphologies of pathogenic microorganisms [45]. 

Using trained CNNs, a large set of image analysis studies have so far have been performed. Smear analysis of various clinical samples by light microscopy is a widely used method for the identification of certain infectious pathogens in the human body. A good example is parasite diagnosis, which can be performed using thick and thin blood smears. A number of AI models have been developed for the automated detection of *Plasmodium* parasites, the causative agent of malaria, which may be highly beneficial in affected regions. CNN using transfer learning has been proposed to automatically detect and quantify *Plasmodium falciparum* at different cellular stages of infection, where diagnostic accuracy is heavily dependent on the expertise of the microscopist [46,47,48,49,50,51,52,53,54]. In their study, Oliveria et al. has applied multilayer perceptron and decision tree as a new approach for detecting malaria parasites in full images of thick blood smears using pixel classifiers. The authors reported precision rates of 91.71% and 93.14% with large parasite sizes, and precision rates of 76.58% and 71.58% were obtained with small parasite sizes [48]. Sengar et al., on the other hand, attempted to automatically detect and classify *Plasmodium vivax* life cycle states for a predictive diagnostic decision and reported the performance of the Vision Transformers (ViTs) model to reach 90.03% accuracy [49]. In a more sophisticated approach, Park et al. investigated the utility of AI application to quantitative phase spectroscopy for the automated analysis for the detection and staging of red blood cells infected with *Plasmodium falciparum* at trophozoite or schizont stage to aid diagnosis. The authors compared various ML techniques, including linear discriminant classification (LDC), logistic regression (LR), and k-nearest neighbor classification (NNC), and they found that LDC provides the highest accuracy of up to 99.7% in detecting schizont stage infected cells, while NNC showed slightly better accuracy (99.5%) than either LDC (99.0%) or LR (99.1%) for discriminating late trophozoites from uninfected RBCs [50]. In a 2023 study, Hemachandran et al. applied neural network models such as CNN, MobileNetV2, and ResNet50 to investigate an automatic image identification system to diagnose malaria blood smears, reporting an accuracy rate of 97.06% of the MobileNetV2 model for disease detection [54]. Interestingly, point-of-care mobile digital microscopy and deep learning methods for the detection of soil-transmitted helminths and *Schistosoma haematobium* has also been looked at in the literature with a reported range of sensitivity 83.3–100% using sequential algorithms [55].

Automated detection algorithms have also been applied to bacterial infections where diagnosis is widely performed using microscopy. Sputum smear microscopy using acid-fast stained slides is the primary and most widely used method for the diagnosis of tuberculosis (TB). *Mycobacterium tuberculosis*, the causative agent of TB, can be observed under the conventional light microscope when examined by experienced personnel; however, this approach is notoriously time-consuming and requires training and expertise. Therefore, computer-aided identification systems represent a promising approach for timely and reproducible results. Candidate detection and classification using CNNs have been proposed for the effective and accurate detection and identification of *M. tuberculosis* in various studies [56,57,58,59,60]. Kuok et al. have utilized a Refined Faster region-based CNN (Faster R-CCN) model for the automated detection of acid-fast bacilli (AFB) detection in smear sputum slides and reported an 86% detection rate of the Faster R-CCN model compared to support vector machine (SVM), which demonstrated a 70.93% overall detection rate [56]. A 2020 study, which focused on a CNN-based active learning framework to identify mycobacteria in digitized Ziehl–Neelsen stained human tissues, used two CNN models, CNN_IN_, CNN_AL_, and reported F1 scores of 99.03% and 98.75%, as well as 99.04% and 98.48% accuracy, respectively, to classify microscopy slides images to be AFB-positive and AFB-negative [57].

The automated interpretation of blood culture Gram stains to identify microorganisms associated with bloodstream infections using a deep CNN has also been investigated by Smith et al. [61]. This study reported the use of 25,488 images from positive blood culture Gram stains for the training of a CNN model, which was reported to demonstrate an accuracy of 94.9% for the automated detection of Gram-positive cocci in chains and pairs and Gram-negative rods. Similarly, an automated detection and segmentation approach has been applied to *Bacillus anthracis*, which causes anthrax, in which neural networks including UNet and UNet++ have been used for the image analysis of tissue slides of patients suffering from the cutaneous anthrax disease to apply detection and segmentation of the bacteria within the digital images with an overall accuracy of 97% [62]. A deep learning method using hyperspectral microscope images was also described for the identification of non-O157 Shiga toxin-producing *Escherichia coli* [63]. On the other hand, ML has also been applied to the analysis of microscopic agglutination tests (MATs) used to diagnose leptospirosis, an infectious disease caused by the pathogenic bacterial species of Leptospira, and it has been suggested to provide an opportunity for the automatization of MATs [64]. In this research, the authors trained an SVM-based ML using MAT images created with sera of Leptospira-infected (positive) and non-infected (negative) hamsters, and they reported a sensitivity and specificity of 0.99 for the confirmed diagnosis. The use of chromogenic agar and colony morphology represent an alternative method for the classification of bacterial species in the clinical microbiology laboratory. Zielinski et al. has demonstrated a method based on deep CNN that obtains image descriptors which are then classified with SVM or random forest to analyze various genera and species of bacteria based on colony morphology stained with Gramm’s method and reported a 97.24 ± 1.07% accuracy of recognition [65]. Similar work was described by Ahmad et al., who employed a deep ensemble approach for pathogen classification in large-scale images using patch-based training and hyper-parameter optimization [66]. The automated detection of *Streptococcus pyogenes* and *Staphylococcus aureus* based on chromogenic agar and AI detection module software has also been described [67,68]. In the study by Gammel et al., the authors compared manual interpretation to Automated Plate Assessment System (APAS Independence) and showed positive and negative percent agreements (PPA and NPA, respectively) to be 100% and 97.3%, respectively. In addition, Rattray et al. investigated the identification of *Pseudomonas aeruginosa* strains from colony image data from clinical and environmental samples for a robust, repeatable detection of phenotype on the level of individual strains, and they reported an average validation accuracy of 92.9% and an average test accuracy of 90.7% for the classification of individual strains [69]. A new few-shot learning method of bacterial colony counting was also described to be useful for *Escherichia coli* on Plate Count Agar (PCA) with YOLOv3 models, aiding colony-forming unit (CFU) counting and bacterial quantification in the clinical laboratory [70].

The ML-assisted automated detection of fungi has also been reported by a number of studies which are mainly based on colony features and microscopic images [71,72,73,74], while ML has been coupled with several different techniques such as Raman spectroscopy, loop-mediated isothermal amplification and transmission electron microscopy for the detection of viruses including SARS-CoV-2 and hepatitis B virus as well as influenza A and B virus in clinical specimens [75,76,77,78,79]. The characteristics of the studies are listed in the table below (Table 3).

While for many years, clinical diagnostic laboratories relied mostly on conventional phenotypic and gene sequencing identification techniques which are time-consuming and labor-intensive, more recently easy, rapid, high-throughput, low-cost and efficient identification techniques such as MALDI-TOF mass spectrometry (MALDI-TOF MS), whole genome sequencing (WGS), multilocus sequence typing (MLST), random amplified polymorphic DNA (RAPD), amplified fragment length polymorphisms (AFLP), pulse field gel electrophoresis (PFGE), and PCR-based replicon typing have been introduced in the field of molecular microbiology and epidemiology. It is worth mentioning that with the increased use of these modern techniques in the last few decades, the application of ML to the interpretation of these techniques has become unavoidable [80,81,82]. Yet, a critical factor for the expansion of AI research in this field is the availability of digital data including genomic and metagenomic data generated using the above-mentioned techniques to all researchers worldwide by the deposition into shared databases.

#### 3.1.3. Automated Factor Analysis

ML approaches have also been applied in the context of clinical sample analysis which may contain variable datasets depending on the infection or no-infection state of the patient. Using this approach, Wang et al. described an automated urine analysis for the increased detection of *Trichomonas vaginalis*, which is a protozoan parasite which causes a common sexually transmitted infection, trichomoniasis. Using classification models constructed using random forest, linear regression and support vector machine, the authors were able to analyze the importance of variables in urine analysis such as levels of nitrite, protein, occult blood, leukocyte esterase, red blood cells, white blood cells and epithelial cells as well as the patient age and gender, and they suggest that the proposed ML-based urine analysis has a high prediction score and can significantly increase the detection rate of T. vaginalis infection in a cost-effective manner [83]. Alternatively, using an XGBoost algorithm, scientists also constructed an ML model for COVID-19 diagnosis based on routine blood parameters including eosinophil count, mean corpuscular hemoglobin concentration (MCHC), albumin, international normalized ratio (INR) and prothrombin activity percentage, and they proposed this model as a diagnostic tool in the clinic [84]. A similar approach has also been used for the differential diagnosis of viral and bacterial meningitis, in which multiple logistic regression (MLR), random forest (RF), and naïve Bayes (NB) algorithms were applied to variables including cerebrospinal fluid (CSF) neutrophil count, CSF lymphocyte count, neutrophil-to-lymphocyte ratio (NLR), blood albumin, blood C-reactive protein (CRP), glucose, blood soluble urokinase-type plasminogen activator receptor (suPAR), and CSF lymphocytes-to-blood CRP ratio (LCR) as predictors. The study indicated that the accuracy for viral and bacterial meningitis should be above 95% and 78%, respectively, in order to obtain optimal predictions of the type of meningitis [85].

#### 3.1.4. Antimicrobial Resistance Analysis

Antimicrobial resistance (AMR) remains one of the most challenging aspects of modern medicine. The application of ML algorithms to the escalating problem of AMR has gained increasing attention in the past 7 years due to the exponential growth of experimental and clinical data, improvements in algorithm performance, investments in computational capacity, and growing urgency for innovative approaches for the treatment of infections due to multidrug-resistant (MDR) microorganisms. In particular, deep learning algorithms have been utilized in the context of predicting antibiotic resistance genes from metagenomic data [86] and genome sequence data [87] as well as the identification of mutations relevant to AMR [88] using traditional ML algorithms and CNN. ML has also been coupled with high-throughput multiplex digital PCR and amplification and melting curve analysis (AMCA), which have been suggested to accurately detect carbapenem-resistance genes (blaIMP, blaKPC, blaNDM, blaOXA-48, and blaVIM) in clinical isolates [89]. Using genome analysis and extreme gradient boosting (XGBoost)-based ML models, scientists have also accurately explored the important genomic regions to predict minimum inhibitory concentrations (MICs) for 15 antibiotics for Salmonella strains [90].

Surface-enhanced Raman spectroscopy (SERS) has been increasingly used for the detection of antibiotic-resistant bacteria due to its various features such as high sensitivity, requirement of a simple sample preparation procedure and low cost. A combination of SERS and deep learning techniques has been used in the discrimination of antibiotic-resistant bacteria including methicillin-resistant *Staphylococcus aureus* (MRSA) and colistin-resistant *Klebsiella pneumoniae* [91,92]. Fu et al. have also reported the possibility of rapid identification of antibiotic sensitivity and MDR patterns among urinary tract pathogens using SERS spectra and CNN [93]. A number of reviews have focused on the application of AI for tackling antibiotic resistance [94,95,96]. Interestingly, a more recent study by Jeon et al. has also explored the use of MALDI-TOF spectral data combined with ML for the identification of MRSA using the MALDI-TOF MS technique. In this study, the authors were able to diagnose MRSA with a sensitivity, specificity and accuracy of 91.8%, 83.3% and 87.6%, respectively [97].

#### 3.1.5. Antimicrobial Discovery

An emerging application of AI is the discovery of novel antimicrobial peptides (AMPs) to tackle the global challenge of AMR. Antibiotic discovery programs mostly depend on large synthetic chemical libraries which contain hundreds of thousands to a few million molecules, and these are typically costly to curate and limited in terms of chemical diversity. Given the recent advancements in ML, this field is now adopting a deep learning approach for the application of molecular property prediction to identify novel classes of antibiotics [98]. With the innovation of modeling neural network-based molecular representations, molecules can be continuously mapped to vectors which are subsequently used to predict their properties. This method provides molecular representation that are highly attuned to the desired property and is far more effective in property prediction accuracy. A concrete example of deep learning-based small molecule discovery is the study by Wang et al. in which various ML models including naïve Bayes, support vector machine, recursive partitioning and k-nearest neighbor algorithm have been used to predict new antimicrobial molecules against *S. aureus* [99]. Another ground-breaking research was reported by Stokes et al., where the authors have leveraged a deep learning architecture called a message-passing neural network (MPNN) to discover structurally novel antimicrobial molecules against *E. coli* by screening ≈ 107 million structurally diverse chemicals [100]. Using this pipeline, the study resulted in the discovery of eight antibacterial compounds that are structurally distant from known antibiotics, among which one compound, halicin, exhibited efficacy against broad-spectrum bacterial infections in vivo. A similar approach was also explored by Liu et al., which resulted in the discovery of a structurally new molecule, abaucin, with antibacterial activity against *Acinetobacter baumanii* [101]. Alternative deep learning-coupled molecular dynamics simulations have also been shown to be effective in the screening and discovery of peptides with antimicrobial activity [102,103]. Alternatively, deep learning techniques such as the long short-term memory (LSTM) generative model and bidirectional LSTM (BiLSTM) classification models have also been employed in the design of novel AMP sequences [104]. These approaches have been systematically reviewed by a number of studies present in the literature [105,106,107].

#### 3.1.6. Microbiome Analysis

With the critical role of the microbiome in various human diseases and the growing importance of microbiome research, characterization of the microbiome and host–microbiome associations remains critical for our understanding of various complex diseases. The omics-based methods, such as metagenomics, metatranscriptomics, and metabolomics, are widely used in the study of gut microbiome due to their ability to provide high-throughput and high-resolution data. The vast amount of data generated via these methods has led to the development of computational methods for data processing and analysis, which is a field where ML can be used as a powerful tool [108]. In this regard, ML provides new insights into the development of models that can be used to predict outputs, such as classification and prediction in microbiology, extrapolation of host phenotypes to predict diseases, and the use of microbial communities to stratify patients by the characterization of state-specific microbial signatures [109]. Advances in this field have been highlighted by several recent studies [110,111,112,113]. 

**Figure 2:**
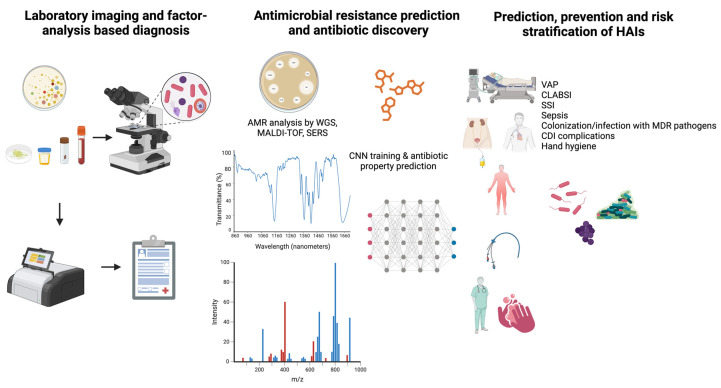
Artificial intelligence applications in the field of microbiology and infectious diseases with a focus on antimicrobial resistance and hospital-acquired infections.

The applicability of AI in the field of microbiology and infectious diseases is summarized in Figure 2.

### 3.2. AI and Hospital-Acquired Infections

Healthcare-associated infections (hospital-acquired infections, HAIs) are nosocomially acquired and are defined as infections that are not present in the patient before hospitalization [114]. HAIs may occur in different wards during treatment and hospital stay, and they are most often associated with hospitalization in intensive care units (ICUs). In ICUs, patients have a 5 to 10 times higher risk of acquiring an HAI due to both intrinsic factors such as immunodeficiency and extrinsic factors such as the administration of medical devices. An ICU is often regarded as the epicenter of microorganisms with MDR [115]. HAIs often occur as a result of using invasive procedures such as the administration of temporary indwelling devices including central venous catheters, urinary catheters, vascular access devices, endotracheal tubes, tracheostomies, enteral feeding tubes, and wound drains or could emerge as a complication after surgical intervention associated with the administration of implants. HAIs comprised a wide range of infections categorized based on infected medical equipment. This includes central line-associated bloodstream infections (CLABSIs) and central venous catheter bloodstream infections (CVCBSIs), catheter-associated urinary tract infections (CAUTIs), and ventilator-associated pneumonia (VAP). The second group covers surgical site infections (SSIs) [116]. Furthermore, *Clostridium difficile* is considered among the most common cause of nosocomial infectious diarrhea with increasing incidence and severity [117]. Hand hygiene has been shown to be the most important risk factor in HAIs [118].

The impact of HAIs is reflected as a considerable clinical and financial burden in terms of prolonged hospital stay, excess death and long-term disability, increased microbial resistance and increased direct costs for the healthcare system [119]. A meta-analyses study estimated the cumulative annual burden of HAIs including CLABSIs, VAP, SSIs, CAUTIs and *C. difficile* infections (CDIs) to be USD 10 billion in the United States [120].

The surveillance of HAIs is a critical component of the implementation and maintenance of effective infection prevention and control programs. HAI surveillance data are generally used for the quantification and monitoring of HAIs burden, detection of outbreaks, identification of risk factors to implement and evaluate control interventions as well as the identification of areas for improvement. HAI surveillance programs enable hospitals to monitor the outcomes of current practice and facilitate the timely feedback to ensure practice improvement and better patient outcomes. ML is a field of AI which allows computers to “learn” and represents an automatic optimization of mathematical models that fits the available data with progressive accuracy [121]. There are two main types of ML: supervised and unsupervised. Supervised learning refers to the use of a training set of data to produce a function that can be utilized to predict a labeled outcome. If the discriminative model does not implement data that have been previously labeled by domain experts, it is termed unsupervised. The application of ML to infection prevention and control is considered as an advantageous approach for an improved understanding of HAI risk factors, enhanced patient risk stratification and identification of transmission routes as well as early detection and control [122]. 

#### 3.2.1. Intensive Care Units (Predictions, Forecasting)

**Figure 3:**
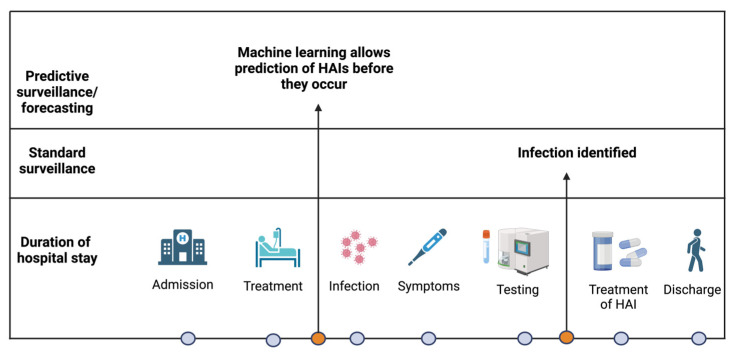
Application of machine learning models to the predictive surveillance and forecasting of hospital-acquired infections (HAIs) (adapted from [125]).

A considerable number of AI and ML models have been developed which can predict the occurrence of an event in advance, which is commonly termed ‘forecasting’ (Figure 3). Considering the excessive number of HAIs occurring in healthcare worldwide, the early detection and possible prevention of HAIs using AI has attracted major attention in the field. AI and ML hold the promise for the development of HAI surveillance algorithms aimed at understanding HAIs risk factors, improvement of patient risk stratification, identification of transmission pathways as well as timely or real-time detection on infections. In this context, electronic health data represent a critical source of information and are increasingly available today. A state-of-the-art data management system provides an opportunity to implement real-time decision support systems for the automated surveillance of HAIs. ML-enabled clinical decision support studies in ICUs focus on ML-supported monitoring and diagnosis, the early identification of clinical events as well as outcome prediction and prognostic assessment followed by treatment decisions to aid clinicians, researchers and policy makers [123,124]. 

So far, an emerging number of ML models have been developed in an effort to forecast VAP, CLABSIs and SSIs as well as the risk of colonization/infection with an MDR pathogen and CDI complications in the hospital setting. However, the forecasting of sepsis and/or septic shock has dominated the field with most studies belonging to this domain.

#### 3.2.2. Ventilator-Associated Pneumonia (VAP)

Ventilator-associated pneumonia (VAP) is defined as pneumonia occurring more than 48 h after a patient has been intubated and received mechanical ventilation [126]. It is considered as the most common nosocomial pneumonia in critically ill patients [127]. The early recognition of patients at a high risk of developing VAP and subsequent prevention of its progression are highly significant in critical care units. Studies have shown that some risk factors are associated with VAP. Although there are some patient-specific risk factors such as age, pre-existing disease (chronic obstructive pulmonary disease, COPD) and a Glasgow Coma Scale (GCS) of 9 or less, also other care-related factors exist such as head-of-the-bed angle, emergency intubation, aspiration, previous antibiotic treatment, and reintubation [128]. A number of studies have focused on the application of ML algorithms for the early prediction of VAP in critical care patients. A recent study by Liang et al. has utilized a random forest algorithm to construct a base classifier for the early discrimination of patients at a high risk of VAP 24 h after intubation. For the analysis, 38,515 ventilation sessions and a set of 42 variables were used, including age, sex, admission source and type, reintubation, pre-existing diseases, the worst value of the partial pressure of the arterial oxygen/fraction of inspired oxygen (PaO_2_/FiO_2_) ratio, white blood cell count (WBC), body temperature in the first 24 h after ventilation, the worst value of the APACHE III and its subcomponents, sequential organ failure assessment (SOFA) and its subcomponents in the first 24 h after admission to the ICU, coma, aspiration, sepsis, bacteremia, trauma/polytrauma, fracture and pneumothorax. Five-fold cross-validation was performed for the evaluation of the model performance. The first five features for the predictive model of VAP were listed as PaO_2_/FiO_2_ ratio, APACHE III score, body temperature, age and WBC followed by admission source and SOFA score. The model was found to achieve an AUC of 84% in the validation, 74% in the sensitivity and 71% in the specificity 24 h after intubation [129]. A similar study was conducted by Giang and colleagues in which the performances of a variety of ML models trained to predict VAP during the patient stay were compared. Electronic health records (EHRs) from 6126 adult ICU encounters lasting at least 48 h following the initiation of mechanical ventilation were included in the study. Data used included a minimum of one observation of each of the following vital signs and lab tests: diastolic blood pressure, creatinine, GCS, heart rate, oxygen saturation (SpO_2_), platelet count, respiratory rate (RR), systolic blood pressure, temperature, hematocrit, and WBC as well as antibiotics, sputum laboratory result, blood culture result, presence of cirrhosis, congestive heart failure, fever, bacteremia, intracranial hemorrhage, renal failure, respiratory distress, respiratory failure, sepsis, subarachnoid hemorrhage, shortness of breath and acute respiratory distress syndrome (ARDS). Among the seven models tested (logistic regression, multilayer perceptron, random forest, support vector machines, XGBoost, CURB-65, PIRO), the highest overall AUROC was demonstrated by XGBoost with an AUROC value of 0.854. The most important features for the best-performing model were indicated to be the length of time on mechanical ventilation, the presence of antibiotics, sputum test frequency, and the most recent GCS assessment [130]. The development of ML models may in the future aid in the implementation of a warning system prior to or during VAP, which can improve diagnosis and prevent both under- and over-treatment with antibiotics.

Similar studies have also been performed to develop a predictive model for individualized risk assessment using ML in order to identify patients at risk of developing VAP [131] and more recently to predict mortality in patients with severe pneumonia who require ICU admission [132,133] as well as the prediction of VAP in defined patient groups such as those with traumatic brain injury [134]. 

#### 3.2.3. Central Line-Associated Bloodstream Infections (CLABSIs)

Central line-associated bloodstream infections (CLABSIs) are defined by the Centers of Disease Control and Prevention (CDC) as laboratory-confirmed bloodstream infections that cannot be attributed to a source other than the presence of a central line and develop 48 h after central line placement [135]. The identification of high-risk patients may be beneficial for clinical practice by enabling earlier or more intensive treatment and monitoring such as the encouragement of more timely replacement of catheters and catheter site dressings. A number of studies have focused on the development of ML algorithms to predict and identify patients at a high risk of developing CLABSIs. In 2022, Rahmani et al. have used data from EHRs of 27,619 patients who utilized a central line procedure and attempted to develop an ML model with the inclusion of variables such as demographics (gender, age, race, ethnicity), the number of days a patient had been hospitalized before placement of a central line, laboratory and vital values (WBC, neutrophil, hemoglobin, temperature), and history of comorbidities (smoking, heart failure, chronic kidney disease (CKD), renal failure, sepsis, valvular disease, diabetes, arrhythmia, presence of a stoma, tumor, cirrhosis, trauma, peptic ulcer disease, peripheral vascular disease). The authors employed three machine learning classifiers, XGBoost, logistic regression and decision tree, to evaluate their performance in terms of AUROC and statistical analysis. In this study, XGBoost was reported to outperform the other two classifiers for the ability to predict CLABSIs with an AUROC of 0.762. The most important subset of features for the CLABSI prediction in the XGBoost model was age, race, temperature, hemoglobin, WBC, neutrophil, and any comorbidity, history of sepsis, chronic kidney disease, stoma, renal failure, valvular disease and previous CLABSI [136]. Similarly, a predictive model was developed based on retrospective data of 70,218 unique patients from a healthcare system which included intrinsic risk factors including age, sex, history of CLABSI, history of immunodeficiency in general, HIV, leukemia, lymphoma, and neutropenia as well as extrinsic risk factors such as chlorhexidine bathing non-adherence (defined as cumulative missed days), routine bathing non-adherence (defined as cumulative missed days), days in hospital prior to placement of the central venous line (CVL), device days of having any CVL, device days for specific CVLs (peripheral, internal jugular, port, femoral, tunneled, or non-tunneled), device days cumulative of all CVLs, and parenteral nutrition. Having tested both models, the authors indicated the random forest model to have a better performance compared to logistic regression and identified age, number of days the patient had any CVL, and the cumulative count of all days the patient had for all CVLs to be the most reliable variables in the prediction of a CLABSI [137]. A comparative predictive analysis has also been reported in literature, in which variables of six different severities of illness scores calculated on the first day of ICU admission with their components and comorbidities from 57,786 patients were included. Predictive models were created for in-hospital mortality, central line placement and CLABSI as outcomes using classifiers including logistic regression, gradient boosted trees and deep learning. This study has proved that the classifier using logistic regression for predicting CLABSI performed with an AUC of 0.722, and the classifier using deep learning had the highest AUC for mortality followed by central line replacement with AUC values of 0.885 and 0.816, respectively [138]. Another recent research group from Boston Children’s Hospital built an ML model for the prediction of impending CLABSIs in 7468 hospitalized cardiac patients and indicated that the model could predict 25% of positive blood cultures with the major predictors being prior history of infection, elevated maximum heart rate, elevated maximum temperature, elevated C-reactive protein, exposure to parenteral nutrition and use of alteplase for central venous catheter (CVC) clearance [139]. These studies among others demonstrate models for identifying patients who will develop CLABSIs and highlight that the early identification of these patients has important implications for quality, cost and outcome improvements. 

#### 3.2.4. Surgical Site Infections (SSIs)

Surgical site infections (SSIs) are among the most common types of postoperative complications associated with substantial morbidity and mortality, prolonged hospital stay, and consequent financial burden to healthcare systems worldwide [140]. Expectedly, several groups have applied ML to create predictive models for SSIs [141,142,143,144,145,146]. In 2021, Petrosyan et al. utilized heath administrative datasets to develop an efficient three-stage algorithm to identify SSIs within 30 days after surgery. In particular, the authors have developed random forest algorithm to perform a preliminary screening of variables followed by the high-performance logistic regression approach to select top 30 most important predictors for SSIs with point system or risk scores. Using datasets including physician procedure claims, hospital (ICD-10) codes and physician (ICD-9) diagnostic codes, the authors were able to demonstrate a high performance of the random forest algorithm for the prediction of SSIs with a high degree of accuracy [147]. In more recent research published in 2023, Wu et al. described the development of an ML model for the detection of SSIs following total hip and knee arthroplasty in which nine XGBoost models were developed and validated to identify incisional SSIs, organ space SSIs and complex SSIs using administrative data and electronic medical records free text data. With data from 16,561 total knee arthroplasty and 10,799 primary total elective hip arthroplasty patients, the authors were able to predict SSIs with XGBoost models achieving a high performance with an ROC AUC of 0.906, and ML models were proposed for the automated detection of complex SSIs [148]. Similarly, Chen et al. utilized perioperative factors from a total of 4019 patients who received a lumbar internal fixation surgery to develop ML models for the prediction of SSI following posterial lumbar spinal surgery. In this study, the authors screened specific variables using logistic regression analysis with three ML algorithms, Lasso regression analysis, support vector machine and random forest, and identified four predictors associated with SSIs including Modic changes, sebum thickness, hemoglobin and glucose levels. By developing a prediction model, the authors reported an ROC AUC value of 0.986 and suggested that the model can help clinicians simplify the monitoring and prevention of SSIs [149]. While research continues, several systems are already in use in healthcare. For instance, the University of Iowa Hospitals & Clinics have utilized ML to reduce surgical site infections by 74% over the past 3 years using Data Analytics for Safe Healthcare (DASH) analytics systems. The hospital is using the high-definition care platform (HDCP) that integrates with the hospital’s EHRs to assess the potential risks and predict SSIs before they occur [150].

#### 3.2.5. Sepsis

In sepsis, the timeliness of detection of an incidence in progress is a crucial factor in the outcome for the patient. As for the AI-aided detection of several infections which occur in the ICU, EHRs can be used as an effective tool for building ML models to improve the timeliness of sepsis detection. So far, a number of studies have incorporated ML models trained from data in individual patient EHRs for the early detection of sepsis [151,152,153,154,155,156]. Further to these studies, Wang et al. extracted and evaluated 55 features from the electronic medical record data of a total of 4449 infected patients, and they applied a random forest algorithm to predict the onset of sepsis. While the authors were able to determine features including neutrophils %, D-Dimer, neutrophils, eosinophils %, lymphocytes %, albumin, WBC, direct bilirubin, potassium, calcium and cholinesterase among others to be of importance in the predictive model of sepsis events, the authors also reported an ROC AUC of 0.91, indicating the good predictive ability of the established ML-based model for sepsis patients in Chinese hospitals [157]. In a separate study, Lauritsen et al. collected retrospective data from multiple Danish hospitals over a seven-year period and compared three different approaches for early sepsis detection: a GB-Vital model, a non-sequential MLP model with thousands of features and a sequential CNN-LSTM model with an equal number of features. In this study, the authors demonstrated a performance ranging from an AUROC of 0.856 3 h before sepsis onset to an AUROC of 0.756 24 h before sepsis onset [158]. Furthermore, AI algorithms have also been developed for the early diagnosis of sepsis in the ICU using real-time data, which have been indicated to have a better performance than SOFA score in sepsis diagnosis, which is commonly regarded as highly sensitive and predictive in the diagnosis of sepsis [159]. Similarly, Fagerström et al. developed a LiSep LSTM; a long short-term memory neural network using 59,000 ICU patient data and showed an AUROC 0.8306 for sepsis prediction [160]. The characteristics of the studies are listed in the table below (Table 4).

#### 3.2.6. *Clostridium difficile* Infection (CDI) and Complications

Despite the imminent burden of HAIs worldwide, the application of ML to this field is still not well explored. In this context, surveillance and patient risk stratification using EHR data remains vital. CDIs represent a prevalent condition which often arises in healthcare settings. In a study based at two large academic health centers, a data-driven approach was undertaken to develop risk prediction models for CDI that work well across institutions. While this study demonstrated that the L2 models achieved AUROC values between 0.75 and 0.82, the authors importantly noted that many of the top predictive factors differed between facilities [161]. Panchavati et al. also performed a comparative analysis of ML approaches to predict *C. difficile* infection in hospitalized patients by incorporating data inputs such as vital signs, laboratory tests, active medication treatment and comorbid medical conditions. Using XGBoost, deep long short-term memory neural network, and one-dimensional convolutional neural network, the authors demonstrated that XGBoost achieved the highest performance with an AUROC of 0.815 for predicting in-hospital CDI after only 6 h of hospital stay [162]. On the contrary, in a study by Escobar et al. evaluating more than 150 potential predictors using multiple techniques including ML, despite a large multi-center cohort and granular electronic medical record data, none of the developed models were able to predict recurrent CDI [163].

Complications of CDI can be listed as ICU admission, development of toxic megacolon, need for colectomy and death. Due to the complexity of treatment regimens for patients with CDI, risk stratification models have so far been investigated. In their study, Li et al. attempted to investigate the applicability of the ML approach for patient risk stratification for complications in adult patients. Using selected patient features including admission details, daily hospitalization details, presence at the hospital location during the 3-day period, current hospitalization information such as high-creatinine flag and metastatic cancer comorbidity, prior CDI, proton pump inhibitor use, Charlson–Deyo Comorbidity Index score, concurrent non-CDI antimicrobial use, solid organ transplant as well as continuous vital signs such as respiratory rate among 1118 cases of CDI, the authors reported an AUROC of 0.69 of the developed model for the diagnosis of CDI complication. The authors reported an improved performance of AUROC 0.90 using data extracted 2 days after CDI diagnosis. This study proposed to accurately stratify CDI cases according to their risk of developing complications [164]. 

#### 3.2.7. Multidrug-Resistant (MDR) Pathogens

Colonization or infection by MDR microorganisms is a major threat for the vulnerable patient population admitted to the ICU. MDR confirmation tests by the microbiology laboratory can take up to 48 h, and hence several AI approaches have been applied to predict risk factors during the first 48 h of ICU admission. In a study by Mora-Jimenez et al. in 2021, the authors considered clinical and demographic features, the use of mechanical ventilation and antibiotics taken by the patients during the time interval. By applying feature selection strategies between MDR and non-MDR patient episodes, the authors were able to define statistically significant features such as SAPS III, Apache II score, age and department of origin for predicting the development of an infection by an MDR pathogen in the ICU [165]. Similarly, in 2022, Liang and colleagues have constructed a carbapenem-resistant Gram-negative bacteria (CR-GNB) carriage prediction model for the ICU. By including 1385 patients with positive CR-GNB cultures and 1525 patients with negative cultures, the authors used 16 variables in the multivariable logistic regression model and built three ML models for all variables included in the study such as demographics, primary disease, comorbidity and clinical characteristics data. The authors reported a better performance of random forest compared to XGBoost and decision tree models. Furthermore, the authors were able to successfully predict 74 cases out of 86 to have a positive CR-GNB culture with an overall accuracy of 85.92%. These studies highlight the possible application of ML models for the prediction of the colonization or infection occurrence within a one-week period in the ICU, guiding medical staff in real time to identify high-risk groups in advance [166].

#### 3.2.8. Hand Hygiene

The hands of healthcare personnel are known to be a primary source of transmission of HAIs, which can be effectively reduced by both practicing hand hygiene (HH) and adhering to HH guidelines [167]. While it can be seen as a simple infection prevention and control strategy, the application of HH can be problematic in the hospital setting. Most studies focusing on HH use the Internet of Things (IoT) to enhance healthcare workers’ self-awareness for HH. A wide range of studies utilized the IoT for monitoring HH, such as the automatic and electronically assisted hand hygiene surveillance system (AHHMS) in hospitals, ICUs, wards and inpatient departments. Among these studies, most of them implemented a cloud-based server and a wearable device (electronic badge, tag or bracelet) with or without automatic feedback (reminder) to improve the HH compliance. In addition, Wi-Fi dispensers and counting systems are also being used in HH improvement [168,169,170,171]. In terms of HH, some studies have attempted to use AI training systems as a cost-effective method in monitoring and improving the quality and quantity of HH [172,173]. These studies have suggested that AI-based training could improve the quality of HH, yet the compliance still remains to be defined. Recently, Greco et al. adopted a CNN to automatically analyze, in real time, the sequence of images acquired by a camera to evaluate the quality of handwashing procedures in healthcare facilities [174]. Similarly, Nagar and colleagues suggested the use of CNN and computer vision to detect an individual’s microorganism level by monitoring their hand-washing technique in an effort to prevent HAIs. Via the analysis of hand movements, the model—which aimed to ensure that each hand wash step was completed according to the WHO guidelines—has been shown to have an accuracy of 96.87% for hand detection, 93.3% for microorganism detection and 85.5% for the compliance system, respectively [175]. The automatic detection of hand hygiene using ML models have also been investigated in further recent studies [176,177,178].

## 4. Future Perspectives

While AI-aided tools are being gradually implemented in the infectious diseases, techniques termed Explainable Artificial Intelligence (XAI) for all fields such as medicine, healthcare, nursing, and engineering have recently emerged [179,180,181]. XAI is based on AI and ML that aim to make the decision-making process of AI systems understandable and interpretable by humans, since the deep learning models are often considered “black boxes” due to their complexity, making it challenging to comprehend how they arrive at specific predictions or decisions. It needs to be pointed out that in the near future, XAI will play a crucial role in the prevention of HAIs in terms of predictive analytics, early detection of HAI, risk stratification, decision support and patient education.

It is important to note that AI-based modeling for the diagnosis and prevention of infections critically depends on the availability of healthcare data that are inherently multimodal, including EHR, personal health records, clinical data, medical images, molecular and multi-omics data. Although there is a plethora of existing shared scientific databases, the main forthcoming of this field is the limited accessibility of genomic, proteomic, metagenomic, and epidemiological data availability generated by researchers. The unavailability of multimodal public data also poses as a limitation that hinders the development of corresponding AI-based research. The leveraging of big healthcare data also requires proper management, storage, and analysis, which imposes hinderances associated with big data handling and digital healthcare with potential impacts on data privacy and security, quality and standardization. 

On the other hand, the expansion of AI systems brings along a number of other issues, among which bias represents a major one. AI-based decision making has the potential to magnify the existing biases and transform new categories and conditions, which have the potential to lead to new types of bias. AI-based research is highly dependent on data quality and completeness, robust reference standards as well as expert interpretation of outputs. Otherwise, errors that are introduced during the ML training process may lead to false negatives, misclassification, or lack of applicability. Depending on how data are collected and the learning algorithms are designed, ML results have the potential to either poorly classify new data (underfitting) or lose the ability to recognize similar patterns in new data (overfitting). AI’s application in healthcare also creates a range of legal issues of transparency, accountability, consensus, and secrecy.

## 5. Conclusions

AI applications are inevitably becoming a part of modern healthcare with a high potential to aid caretakers and decision-makers in the fields of laboratory and imaging diagnosis, antimicrobial stewardship, discovery of antimicrobials, microbiome-based translational interventions, infectious disease surveillance, prediction and prevention. The mass digitalization of health records making data accessible and advances in computer power has been instrumental and will remain crucial for future research and development in the field. Although AI is commonly regarded as a threat for “common” jobs, its integration into healthcare should instead be seen as an opportunity for improved patient care and infection management, increased survival, better allocation of staff and resources and lowered costs in healthcare systems.

## Figures and Tables

**Table 1:** Application of artificial intelligence to infectious disease research and management, approaches and challenges.

Application of AI in Infectious Diseases	Approaches	Challenges
Laboratory and imaging diagnosis	Digital culture plate reading Pathogen detection and identification via microscopy images Analysis of RT-PCR data Analysis of MALDI-TOF MS, SERS spectra Clinical radiography imaging analysis Feature/factor analysis of clinical laboratory data	Data standardization among laboratories and centers Availability of big data Data quality and management Risk of bias Legal issues
Antimicrobial resistance analysis	Detection of MDR pathogens Antimicrobial susceptibility analysis Analysis of genomic, sequencing and spectral data	
Antimicrobial discovery	Molecule screening Chemical library mining Design of antimicrobial peptides	
Microbiome analysis	Mining of metagenomic, metatranscriptomic and metabolomic data	
Clinical decision support	Infection prediction and risk stratification Tracking infection epidemiology Tracking human behavior and adherence	

**Table 2:** Summary of the included studies concerning the use of AI in the field of COVID-19 clinical laboratory and radiology detection.

Authors (Year)	*n*	Diagnosis Method	Input	Model/Analysis	Objective
Alouani et al. [7] (2021)	50,146	Real-time PCR (RT-PCR)	Fluorescent readings	Deep convolutional neural network-based software (qPCRdeepNet) https://github.com/davidalouani/qPCRdeepNet, accessed date (18 February 2024)	Detection of false positive results and improvement of test specificity, a quality assurance tool
Lee et al. [8] (2022)	5810	Real-time PCR (RT-PCR)	Fluorescence values	Long-term short memory (LSTM)	Improvement of the speed of COVID-19 RT-PCR diagnosis
Özbilge et al. [9] (2022)	560	Real-time PCR (RT-PCR)	Amplification curves	MobileNetV2 DCNN	Rapid and reliable diagnosis
Villarreal-González et al. [10] (2020)	14,230	RT-PCR	RT-PCR curves	K-neighbor classifier, support vector machine for classification (SVC), decision tree classifier, random forest classifier (RFC)	Detecting atypical profiles in PCR curves caused by contamination or artifacts
Alvargonzález et al. [11] (2023)	20,418	rRT-PCR	Ct values	Support vector machine (SVM) and neural network (NN)	Detection of a Ct pattern that is characteristic of virus variants
Beduk et al. [12] (2022)	63	Laser-scribed graphene (LSG) sensors coupled with gold nanoparticles (AuNPs)	Electrochemical sensor data	Dense neural network (DNN)	Utilization of point-of-care device as biosensing platform for new variants
Tschoellitsch et al. [13] (2021)	1357	SARS-CoV-2 RT-PCR test and blood tests	RT-PCR and blood tests results	Random forest algorithm	Prediction of SARS-CoV-2 PCR results with routine blood tests
Brinati et al. [14] (2020)	279	Routine blood tests and COVID-19 RT-PCR tests	Blood test parameters and COVID-19 RT-PCR test results	Decision tree (DT); extremely randomized trees (ETs), k-nearest neighbor (KNN) Logistic regression (LR), naïve Bayes (NB), random forest (RF), support vector machine (SVM)	Discrimination between SARS-CoV-2 positive and negative patients
Yang et al. [15] (2020)	3,356	Routine blood tests, COVID-19 RT-PCR tests	Blood parameters, COVID-19 RT-PCR test results	Gradient boosting decision tree (GBDT), random tree (RT), logistic regression (LR), decision tree (DT)	Diagnosis of COVID-19 using the results of routine laboratory tests
Abayomi-Alli et al. [16] (2022)	279	Routine blood tests	Hematochemical values	KNN, linear SVM, RBF SVM, random forest, decision tree, neural network (multilayer perceptron), AdaBoost, extremely randomized trees (ExtraTrees), naïve Bayes, LDA, QDA, logistic regression, passive classifier, ridge classifier, and stochastic gradient descent classifier (SGDC)	Effective detection of COVID-19 using routine laboratory blood test results
Rocca et al. [17] (2020)	311	MALDI-TOF MS AND RT-PCR	Main spectra profiles	ClinPro Tools, GA/k-nearest neighbor algorithm	Identification of biomarker patterns for COVID-19
Le et al. [18] (2023)	200	LC/MS-MS	Mass spectra	SHapley Additive exPlanations (SHAP), gradient boosted decision trees, scikit-learn v0.23.2 for random forest, stratified k-fold cross-validation, grid search	Development of an alternative diagnostic strategy for SARS-CoV-2 diagnosis
Rosado et al. [19] (2021)	550	Multiplex serological assay, RT-PCR	IgG and IgM antibody responses, RT-qPCR results	Random forest algorithm	Development of accurate serological diagnostics
Nachtigall et al. [20] (2020)	3621	MALDI-MS, RT-PCR	Mass spectra	Decision tree, DT; k-nearest neighbors, KNN; naive Bayes, NB; random forest, RF; support vector machine with a linear kernel, SVM-L; support vector machine with a radial kernel, SVM-R)	Alternative detection of SARS-CoV-2 in nasal swabs
Costa et al. [21] (2022)	360	MALDI-TOF MS	Mass spectra	Support vector machine with linear kernel (SVM-LK), support vector machine with radial basis function kernel (SVM-RK), random forest (RF) and k-nearest neighbors (K-NN), and linear discriminant analysis (LDA)	Alternative method for detection of SARS-CoV-2 in nasal swabs
de Fátima Cobre et al. [22] (2022)	192	LC-MS	Mass spectra	PLS-DA, ANNDA, XGBoostDA, SIMCA, SVM, LREG and KNN	Prediction of COVID-19 diagnosis, severity, and fatality
Ikponmwoba et al. [23] (2022)	20	SERS	Spectra	Gaussian process classifier (GPC), k-fold cross-validation	Predictive diagnosis of COVID-19 in biological samples

**Table 3:** The characteristics of the studies included regarding the application of machine learning to image analysis in clinical microbiology laboratory.

Authors (Year)	*n*	Diagnosis Method	Input	Model/Analysis	Objective
Loh et al. [46] (2021)	297	Blood smear microscopy	Microscopic smear images	Mask R-CNN	Alternative method for automated rapid malaria screening
Holmström et al. [47] (2020)	125	Thin blood and Giemsa-stained thick smear microscopy	Microscopic smear images	Cloud-based machine-learning platform (Aiforia Cloud and Create), GoogLeNet network	Digitalization of blood smears, application of deep learning (DL) algorithms to detect *Plasmodium falciparum*
Oliveira et al. [48] (2022)	676	Thick blood smear films	Microscopy images	Multilayer perceptron (MLP) and decision tree (DT)	Automated malaria diagnosis
Sengar et al. [49] (2022)	2329	Thin blood smears	Microscopic images	Generative adversarial network (GAN), Vision Transformers (ViTs)	Automated, non-invasive multi-class *Plasmodium vivax* life cycle classification and malaria diagnosis
Park et al. [50] (2016)	413	Quantitative phase spectroscopy	Quantitative phase images of unstained cells	Linear discriminant classification (LDC), logistic regression (LR), and k-nearest neighbor classification (KNC),	Automated analysis for detection and staging of red blood cells infected with *Plasmodium falciparum* at trophozoite or schizont stage
Kassim et al. [51] (2021)	5972	Thick smear films	Annotated thick smear microscopy images	Mask regional–convolutional neural network (Mask R-CNN), ResNet50 classifier	Application of PlasmodiumVF-Net for automated malaria diagnosis on both image and patient level
Dey et al. [52] (2021)	27,558	Thick blood films	Blood smear cell microscopy images	ResNet 152 model integrated with the deep greedy network	Automating the detection of malaria parasites in thin blood smear images
Ufuktepe et al. [53] (2021)	955	Thin blood smears	Thin blood smear microscopy	Channel-wise feature pyramid network for medicine (CFPNet-M)	Red blood cell detection, counting infected cells or identifying parasite species
Hemachandran et al. [54] (2023)	27,558	Blood smears	Blood smear microscopy images	CNN, MobileNetV2, and ResNet50	Automatic image identification system for parasite-infected RBC detection
Holmström et al. [55] (2017)	7385	Iodine-stained stool sample smears	Digital images from a mobile microscope and whole slide-scanner	Sequential algorithms	Automated detection of soil-transmitted helminths and *Schistosoma haematobium*
Kuok et al. [56] (2019)	19,234	Sputum smears stained by acid-fast staining	Smear microscopy images	Refined Faster region-based CNN, support vector machine (SVM)	Two-stage *Mycobacterium tuberculosis* identification system
Yang et al. [57] (2020)	167	Ziehl–Neelsen stained human tissue samples	Digitized images	CNN_IN_, CNN_AL_	Automated identification of mycobacteria in human tissues
Ibrahim et al. [58] (2021)	1050	Acid-fast staining of sputum	Microscopy images	AlexNet model	Automated detection of *Mycobacterium tuberculosis* using transfer learning
Xiong et al. [59] (2018)	3,088,492	Acid-fast stained tissue samples	Microscopy images	CIFAR-10 CNN	AI-assisted detection method for acid-fast stained TB bacillus
Horvath et al. [60] (2020)	15,204	Auramine-stained sputum smears	Slide microscopy images	DNN classifier, Keras, TensorFlow	Machine-assisted interpretation of auramine stains for microscopic tuberculosis diagnosis
Smith et al. [61] (2018)	25,488	Gram staining of blood cultures	Microscopy images	Inception v3 CNN, Python, TensorFlow	Automated interpretation of blood culture gram stains
Hoorali et al. [62] (2020)	954	Tissue slides of patients suffering from cutaneous anthrax	Microscopy images	UNet and UNet++, Keras, TensorFlow	Automatic and rapid diagnosis of anthrax via detection and segmentation of *Bacillus anthracis*
Kang et al. [63] (2020)	84,000	Hyperspectral microscope imaging (HMI) method	Hyperspectral microscope images	Linear discriminant analysis (LDA), support vector machine (SVM) and soft-max regression (SR)	Identification of non-O157 Shiga toxin-producing *Escherichia coli* (STEC) using deep learning
Oyamada et al. [64] (2021)	910	Microscopic Agglutination Test (MAT)	MAT microscopic images	Support vector machine (SVM)	Determine agglutination within microscopic images for the diagnosis of leptospirosis
Zieliński et al. [65] (2017)	660	Stained clinical samples	DIBas dataset of digital bacterial images	CNN, support vector machine, random forest	Deep learning-based classification of bacterial genera and species
Ahmad et al. [66] (2023)	480	Stained clinical samples	High-resolution microscopic images from DIBas dataset	InceptionV3, MobileNetV2	Deep ensemble approach-based pathogen classification in large-scale images
Van et al. [67] (2019]	480	Clinical throat specimens on CHROMagar confirmed by MALDI-TOF MS	Microscopic images	WASPLab PhenoMATRIX chromogenic detection module	AI-detection of *Streptococcus pyogenes* using CHROMagar
Gammel et al. [68] (2021)	5913	Patient samples collected from the nares plated onto BD BBL CHROMagar MRSA II and BD BBL CHROMagar *Staph aureus*	Digital images	Automated Plate Assessment System (APAS Independence)	Evaluation of an automated plate assessment system
Rattray et al. [69] (2023)	335	Culture specimens of clinical and environmental *P. aeruginosa* isolates	Digital colony images	ResNet-50, VGG-19, MobileNetV2 and Xception	Identification of from colony image data
Zhang et al. [70] (2022)	960	*Escherichia coli* cultures on agar medium	Digital colony images	Random cover targets algorithm (RCTA), YOLOv3	Deep learning-based bacterial colony detection
Koo et al. [71] (2021)	3707	Slides with skin and nail specimens	Microscopy images	YOLO v4	Automated detection of superficial fungal infections
Ma et al. [72] (2021)	17,142	Dissecting microscopy (DM)/stereomicroscopy platform	Original colony images	Xception	Validating a novel approach for the detection of Aspergillus fungi via stereomicroscopy
Liu et al. [73] (2015)	1000	Fecal specimens	Microscopic fecal images	ANN-1, ANN-2	Automatic identification of fungi in fecal specimens
Meeda et al. [74] (2019)	30	Fungal cultures, confocal microscopy	Colony fingerprint digital images	Support vector machine (SVM) and random forest (RF)	Rapid discrimination of fungal species by the colony fingerprinting
Khan et al. [75] (2018)	119	Raman spectroscopy	Spectral images	Support vector machine (SVM)	Analysis of hepatitis B virus infection in blood sera using ML
Rohaim et al. [76] (2020)	199	Reverse-transcribed loop-mediated isothermal amplification (LAMP) assay	Quantitative measurements using qRT-PCR	CNN model with binary cross-entropy and Adam	Rapid detection of SARS-CoV-2 using AI in loop-mediated isothermal amplification assays
Ito et al. [77] (2018)	35	Transmission electron microscopy (TEM)	Microscopy images	Cross-point method (CPM), RDP, spectral rings (SR), fully convolutional neural networks (FCN and FCN+)	Automated feline calicivirus particle detection in TEM images
Tong et al. [78] (2019)	600	Raman spectroscopy of serum samples	Raman spectra	Principal component analysis (PCA), support vector machine (SVM)	AI-aided detection of hepatitis B virus infection using Raman spectroscopy
Tabarov et al. [79] (2022)	90	Surface-enhanced Raman scattering spectroscopy (SERS)	SERS spectra	Support vector machine (SVM)	Detection of A and B influenza viruses by SERS coupled with ML

**Table 4:** The characteristics of the studies are included concerning the application of artificial intelligence algorithms to the prediction, surveillance and prevention of hospital-acquired infections.

Authors (Year)	Dataset	Input	Model/Analysis	Objective	Results
Liang et al. [129] (2022)	Multiparameter Intelligent Monitoring in Intensive Care (MIMIC)-III dataset	42 VAP risk factors at admission and routinely measured the vital characteristics and laboratory results from 38,515 ventilation sessions	Random forest compared to clinical pulmonary infection score (CPIS)-based model	Early prediction of ventilator-associated pneumonia in critical care patients	AUC of 84% in the validation, 74% sensitivity and 71% specificity 24 h after intubation
Giang et al. [130] (2021)	Multiparameter Intelligent Monitoring in Intensive Care (MIMIC)-III dataset	Data from 6126 adult ICU encounters	Five different ML models trained: logistic regression, multilayer perceptron, random forest, support vector machines, and gradient boosted trees	Prediction of ventilator-associated pneumonia with ML	The highest performing model achieved an AUROC value of 0.854
Samadani et al. [131] (2023)	Philips eRI dataset	9204 presumed VAP events	XGBoost gradient boosting algorithm, random forest, logistic regression, ADABoost, KNN	Early prediction and hospital phenotyping of ventilator-associated pneumonia	The model predicts the development of VAP 24 h in advance with an AUC of 76% and AUPRC of 75%
Jeon et al. [132] (2023)	SNU-SMG Boramae Medical Center database	816 patient data including the period from hospital admission to ICU admission, age, APACHE II scores, PaO_2_/FiO_2_ ratio, history of chronic respiratory disease, history of cerebrovascular accident (CVA) or dementia, mechanical ventilation, use of vasopressors	Logistic regression with L2 regularization, gradient-boosted decision tree (LightGBM), multilayer perceptron (MLP)	ML-based prediction of in-ICU mortality in pneumonia patients	ML models significantly outperformed the Simplified Acute Physiology Score II (AU-ROC: 0.650 vs. 0.820 for logistic regression vs 0.827 for LightGBM 0.838 for MLP
Wang et al. [133] (2023)	MIMIC-IV and eICU databases	MIMIC-IV (*n* = 4697) and eICU (*n* = 13,760) databases, six variables included: metastatic solid tumor, Charlson Comorbidity Index, readmission, congestive heart failure, age, and Acute Physiology Score II	Logistic regression, decision tree, random forest, multilayer perceptron, XGBoost	Prediction of mortality in pneumonia patients on intensive care unit admission	AUC value ranged in predicting 1-year and hospital mortality were 0.784–0.797 and 0.691–0.780, respectively
Wang et al. [134] (2023)	Medical Information Mart for Intensive Care-III (MIMIC-III) database	786 VAP incidences with traumatic brain injury (TBI) patients	Random forest, XGBoost and AdaBoost	Development of algorithms for prediction of ventilator associated pneumonia in traumatic brain injury patients	The random forest performed the best on predicting VAP in the training cohort with AUC of 1.000. AdaBoost performed the best on predicting VAP in the validation cohort with a AUC of 0.706.
Rahmani et al. [136] (2022)	National longitudinal electronic health records	Demographics, number of days a patient had been hospitalized before placement of a central line, laboratory and vital values (*n* = 27,619)	XGBoost, logistic regression, decision tree	Early prediction of central line associated bloodstream infection using ML	XGBoost was the highest performing model with an AUROC of 0.762 for CLABSI risk prediction at 48 h after the recorded time for central line placement
Beeler et al. [137] (2018)	Indiana University Health Academic Health Center (IUH AHC) database	Intrinsic and extrinsic risk factors (*n* = 70,218)	Logistic regression and random forest	ML-based assessment of patient risk for central line-associated bacteremia	Random forest had AUROC of 0.82, while AUROC curve for the logistic regression model was 0.79
Parreco et al. [138] (2018)	Multiparameter Intelligent Monitoring in Intensive Care III database	Variables included six different severities of illness scores calculated on the first day of ICU admission with their components and comorbidities. The outcomes of interest were in-hospital mortality, central line placement, and CLABSI (*n* = 57,786)	Logistic regression, gradient boosted trees, and deep learning.	Prediction of central line-associated bloodstream infections and mortality using supervised ML	Classifiers using deep learning performed with the highest AUC for mortality, 0.885 and central line placement, 0.816. The classifier using logistic regression for predicting CLABSI performed with an AUC of 0.722
Bonello et al. [139] (2022)	Boston Children’s Hospital database	Patient-level risk factors, encounter-level risk factors, demographics, vital signs measurements from the preceding 24 h, recent course-related risk factors, laboratory values and CVC-associated risk factors (*n* = 7468)	Generalized linear modeling, random forest, lasso regression	Prediction of impending CLABSI infections in hospitalized cardiac patients	ML predicted 25% of patients with impending CLABSI with an FPR of 0.11% and AUC of 0.82
Hu et al. [141] (2015)	Surgical patient database at the University of Minnesota Medical Center	Clinical data included six data types: demographics, diagnosis codes, orders, lab results, vital signs, and medications. Demographics included each patient’s gender, race, and age at the time of surgery	Single-task learning, Hierarchical classification, offset method, propensity-weighted observations (PWO), multi-task learning with penalties (MTLP), partial least squares regression (PLS)	Automated detection of postoperative complications using EHR data	The models demonstrated high detection performance, which ensures the feasibility of accelerating manual chart review (MCR)
Kuo et al. [142] (2018)	Kaohsiung Chang Gung Memorial Hospital database	Dataset including 1836 patients with 1854 free-flap reconstructions and 438 postoperative SSIs	Feed-forward artificial neural network (ANN) and logistic regression (LR) models	Artificial neural network approach to predict surgical site infection after free-flap reconstruction in patients receiving surgery for head and neck cancer	ANN had a significantly higher AUC (0.892) of postoperative prediction and AUC (0.808) of pre-operative prediction than LR
Sohn et al. [143] (2017)	American College of Surgeons National Surgical Quality Improvement Program (ACS-NSQIP) cohort	Cohort data	Bayesian network coupled with natural language processing (NLP)	Detection of clinically important colorectal surgical site infection	Bayesian network detected ACS-NSQIP-captured SSIs with a receiver operating characteristic AUC of 0.827
Soguero-Ruiz et al. [144] (2015)	EHR of the Department of Gastrointestinal Surgery at the University Hospital of North Norway	A cohort based on relevant International Classification of Diseases (ICD10) or NOMESCO Classification of Surgical Procedures (NCSP) codes related to severe post-operative complications (101 cases and 904 controls)	Gaussian process (GP) regression, support vector machine (SVM)	Data-driven temporal prediction of surgical site infection	Real-time prediction and identification of patients at risk for developing SSI was shown
Mamlook et al. [145] (2023)	American College of Surgeons’ National Surgical Quality Improvement Program (ACS NSQIP) database	Data from 2,882,526 surgical procedures	Logistic regression (LR), naïve Bayes (NB), random forest (RF), decision tree (DT), support vector machine (SVM), artificial neural network (ANN), and deep neural network (DNN)	Prediction of surgical site infections using patient pre-operative risk and surgical procedure factors	DNN model offers the best predictive performance with 10-fold compared to the other 6 approaches considered (area under the curve 0.8518, accuracy 0.8518, precision 0.8517, sensitivity 0.8527, F1-score 0.8518)
Cho et al. [146] (2023)	Samsung Medical Center clinical data warehouse (CDW)	Clinical data	Python, Tensorflow, Keras, Scikit-learn libraries, random forest (RF), gradient boosting (GB), and neural network (NN) with or without recursive feature elimination (RFE)	Development of ML models for the surveillance of colon surgical site infections	NN with RFE using 29 variables had the best performance with an AUC of 0.963. PPV of 21.1%, sensitivity of 95%
Petrosyan et al. [147] (2021)	The Ottawa hospital database	Patients aged 18 years and older who underwent surgery, included in the American College of Surgeons National Surgical Quality Improvement Program (NSQIP) data collection	Random forest algorithm, high-performance logistic regression	Prediction of postoperative surgical site infection with administrative data	Final model, including hospitalization diagnostic, physician diagnostic and procedure codes, demonstrated excellent discrimination (C statistics, 0.91, 95% CI, 0.90–0.92
Wu et al. [148] (2023)	Calgary, Canada acute care hospital database	Cohort included adult patients (age ≥ 18 years) who underwent primary total elective hip (THA) or knee (TKA) arthroplasty	XGBoost models	ML-aided detection of surgical site infections following total hip and knee arthroplasty	XGBoost models using a combination of administrative data and text data to identify complex SSIs achieved the best performance, with F1 score of 0.788, ROC AUC of 0.906
Chen et al. [149] (2023)	The First Affiliated Hospital of Guangxi Medical University, Department of Spine and Osteopathy Ward database	Patients who underwent lumbar internal fixation surgery at (*n* = 4019)	Lasso regression analysis, support vector machine, random forest	Application of ML to predict surgical site infection after lumbar spine surgery	C-index of the model was 0.986, ROC AUC curve 0.988
Wang et al. [157] (2021]	Observational cohort from the Intensive Care Unit of the First Affiliated Hospital of Zhengzhou University	Electronic medical record data, a set of 55 features (variables) from 4449 infected patients	Random forest	Application of ML for accurate prediction of sepsis in ICU patients	ROC AUC was 0.91 with 87%, sensitivity, 89% specificity for sepsis prediction
Lauritsen et al. [158] (2020)	Retrospective data from multiple Danish hospitals	EHR, including biochemistry, medicine, microbiology, medical imaging, and the patient administration system (PAS)	Combination of a convolutional neural network and a long short-term memory network	Early detection of sepsis utilizing deep learning on EHR event sequences	Model performance ranged from AUROC 0.856 (3 h before sepsis onset) to AUROC 0.756 (24 h before sepsis onset)
Yuan et al. [159] (2020)	Prospective open-label cohort study conducted at Taipei Medical University Hospital	Data including the vital signs, laboratory results, examination reports, text data, and image of every ICU patient	Logistic regression, support vector machine, XGBoost, and neural network	Development an AI algorithm for early sepsis diagnosis in the intensive care unit	Established AI algorithm achieved accuracy of 82%, sensitivity of 65%, specificity of 88%, precision = 67%, F1 = 0.66 ± 0.02. AUROC was 0.89
Fagerström et al. [160] (2019)	Medical Information Mart for Intensive Care database	Vital signs, laboratory data, and journal entries (*n* = 59,000 ICU patients)	LiSep LSTM; a long short-term memory neural network, Keras with a Google TensorFlow	Application of ML algorithm for early detection of septic shock	LiSep LSTM outperforms a less complex model, using the same features and targets, with an AUROC 0.8306

## Data Availability

All the articles and data sources reviewed in this systematic review are publicly available online. Readers can access the full-text articles and associated data through the respective journal websites, databases, or repositories where they were originally published or hosted.

## Abbreviations

AFB	acid-fast bacilli
AFLP	amplified fragment length polymorphisms
AHHMS	automatic and electronically-assisted hand hygiene surveillance system
AI	artificial intelligence
ALP	alkaline phosphatase
ALT	alanine transaminase
AMCA	amplification and melting curve analysis
AMP	antimicrobial peptide
AMR	antimicrobial resistance
APAS	automated plate assessment system
ARDS	acute respiratory distress syndrome
AST	aspartate aminotransferase
AuNPs	gold nanoparticles
AUROC	area under the receiver operator curve
BiLSTM	bidirectional long-term short memory
CAUTI	catheter-associated urinary tract infection
CDC	Centers of Disease Control and Prevention
CDI	*C. difficile* infection
CFPNet-M	Channel-wise Feature Pyramid Network for Medicine
CFU	Colony-forming unit
CKD	chronic kidney disease
CLABSI	central line-associated bloodstream infection
CNN	convolutional neural network
COPD	chronic obstructive pulmonary disease
CPM	cross-point method
CR-GNB	carbapenem-resistant Gram-negative bacteria
CRP	C-reactive protein
CSF	cerebrospinal fluid
CT	computed tomography
CVC	central venous catheter
CVCBSI	central venous catheter bloodstream infection
CVL	central venous line
CX-R	chest X-ray
DASH	data analytics for safe healthcare
DNN	dense neural network
DT	decision tree
ET	extremely randomized trees
Faster R-CCN	Faster region-based CNN
FCN	fully convolutional neural network
GAN	generative adversarial network
GCS	Glasgow Coma Scale
GGT	gamma-glutamyl transferase
GPC	Gaussian process classifier
HAI	hospital-acquired infection
HDCP	high-definition care platform
HER	electronic health records
HH	hand hygiene
ICU	intensive care unit
IoT	Internet of Things
KNN	k-nearest neighbors
LC/MS-MS	liquid chromatography with tandem mass spectrometry
LCR	lymphocytes-to-blood CRP ratio
LDA	linear discriminant analysis
LDC	linear discriminant classification
LDC	linear discriminant classification
LDH	lactate dehydrogenase
LR	logistic regression
LR	logistic regression
LSP	laser-scribed graphene
LSTM	long short-term memory
MALDI-TOF	matrix-assisted laser desorption ionization–time of flight mass spectrometry
Mask R-CNN	mask regional-convolutional neural network
MAT	microscopic agglutination tests
MCHC	mean corpuscular hemoglobin concentration
MDR	multidrug resistant
MIC	minimum inhibitory concentration
MIMIC	Multiparameter Intelligent Monitoring in Intensive Care
ML	machine learning
MLP	multilayer perceptron
MLR	multiple logistic regression
MLST	multilocus sequence typing
MODE	multi-objective differential evolution
MPNN	message-passing neural network
MRSA	methicillin-resistant *Staphylococcus aureus*
NB	naïve Bayes
NLP	natural language processing
NLR	neutrophil-to-lymphocyte ratio
NN	neural network
NNC	nearest neighbor classification
NPA	negative percent agree
PaO_2_/FiO_2_	partial pressure of the arterial oxygen/fraction of inspired
PCA	plate count agar
PFGE	pulse field gel electrophoresis
PPA	positive percent agree
PRISMA	Preferred Reporting Items for Systematic Reviews and Meta-Analyses
RAPD	random amplified polymorphic DNA
RCTA	random cover targets algorithm
RF	random forest
RFE	recursive feature elimination
RT–PCR	reverse transcriptase polymerase chain reaction
SARS-CoV-2	severe acute respiratory syndrome coronavirus 2
SERS	surface-enhanced Raman scattering
SGDC	stochastic gradient descent classifier
SHAP	SHapley Additive exPlanations
SOFA	sequential organ failure assessment
SpO_2_	oxygen saturation
SR	soft-max regression
SSI	surgical site infection
suPAR	blood-soluble urokinase-type plasminogen activator receptor
SVC	support vector machine for classification
SVM-LK	support vector machine with linear kernel
SVM-RK	support vector machine with radial basis function kernel
SVM	support vector machine
TB	tuberculosis
VAP	ventilator-associated pneumonia
ViTs	Vision Transformers
WBC	white blood cell count
WGS	whole genome sequencing
XAI	explainable artificial intelligence
XGBoost	extreme gradient boosting
